# Response to Cohen et al

**DOI:** 10.1097/PR9.0000000000000731

**Published:** 2019-04-16

**Authors:** Rae F. Bell, Eija A. Kalso

**Affiliations:** a Regional Centre of Excellence in Palliative Care, Haukeland University Hospital, Bergen, Norway; b Perioperative, Intensive Care and Pain Medicine, Pain Clinic, Helsinki University Hospital, University of Helsinki Helsinki, Finland

We thank Drs. Cohen, Schwenk, and Viscusi for their letter with information about the recently published guidelines on the use of ketamine for acute pain and chronic noncancer pain.^1^ We commend the American Society of Regional Anesthesia & Pain Medicine and the American Academy of Pain Medicine for this important initiative. We trust that the guidelines will be regularly updated, in accordance with best current evidence and clinical expertise.

## Disclosures

The authors have no conflict of interest to declare.

## References

[1] Cohen SP Schwenk ES Viscusi ER Ketamine for pain management: let's not neglect practical concerns. PAIN Reports 2019;4:e728. 10.1097/PR9.0000000000000728 PMC6749911 31583345

